# Fluid Extracts

**Published:** 1869-08-15

**Authors:** 


### *Fluid Extracts*

Our thanks are due Messrs Chapman & Dunks (see advertisement) for very excellent samples of Fluid Extracts, aconite, cimicifuga, ergot, hyoscyamus and veratrum viride. The efficient qualities of each are preserved with a degree of perfection we have not known surpassed.

